# Orality and overtness: effects on Spanish subject use

**DOI:** 10.1515/jhsl-2024-0017

**Published:** 2025-07-31

**Authors:** Gemma McCarley

**Affiliations:** University of Toronto, Toronto, Canada

**Keywords:** subject pronoun expression, orality, Latin American Spanish, corpus linguistics, diachrony

## Abstract

This study of a corpus of varieties of Spanish finds that the level of orality of a text is a strong predictor of subject pronoun expression. Following previous studies’ application of orality to interrogative constructions in Brazilian Portuguese and French, an orality measurement was adapted for Spanish and applied to the new corpus *Corpus Diacrónico del Español Latinoamericano: Edición de Sujetos* (CorDELES). CorDELES was created to investigate the historic development of subject pronoun expression that led to the high rates of overt subject pronouns attested in current varieties of Latin American Spanish, specifically whether overt subject pronoun expression increases following contact with the enslaved Africans brought to the Caribbean during the colonial period. This contact hypothesis was used as a backdrop to investigate the effects of orality on a corpus. Indeed, the inclusion of orality as a predictor in a mixed-effects model found significant effects for a distinction between Spain and the Americas as well as an intriguing interaction between year and orality. These results add to the burgeoning body of work revealing the benefits of accounting for orality in corpus work.

## 1 Introduction

When compiling a written corpus, it is important to account for textual differences, as the effects of genre, register, and text-type are well-known (cf. Biber 2001; Biber and Conrad 2019; Szmrecsanyi 2006). However, these kinds of static categorizations do not account fully for stylistic variation and change. Orality, on the other hand, is an independent metric that often corresponds with the level of formality expressed in certain genres but is not beholden to them. Indeed, changes in orality levels within genres have already been shown to create the illusion of ‘apparent’ change in historical corpora. Specifically, this factor has been applied to *wh*-interrogatives in Brazilian Portuguese (BP) (Rosemeyer 2019), and clefted questions in French (Mazzola et al. 2023; Rosemeyer 2022), but not yet to the phenomenon of subject pronoun expression (SPE). Given the influence differing spoken genres have had on SPE rates in Spanish (cf. Travis 2007), the context of variable SPE in Latin American Spanish seems a prime testing ground.

In the last fifty years, the study of SPE in Spanish has become an increasingly popular topic. Variationist research has swelled, producing quantitative and qualitative studies of SPE in the Caribbean (Alfaraz 2015; Camacho 2013; Orozco and Guy 2008; among many others), Central America, South America (e.g. Cerrón-Palomino 2018; Travis 2005), and the U.S. (Otheguy and Zentella 2007). The major throughline of this boom has been the observation that varieties of Spanish spoken throughout Latin America, most notably in the Caribbean, use overt subject pronouns to a higher degree and in more contexts than standard Latin American or Peninsular Spanish. Despite many dialectal differences, such as variation in the pronominal subject system (e.g. the use of *usted* [you.SG.F]; the loss of *vosotros* [you.PL.INF] outside of Spain; and the *vos* [you.SG.INF] form in parts of Latin America [(Real Academia Española)]), each standard Spanish only allows overt pronouns when they mark emphasis or focus. Yet the Dominican example (1) below shows an unmarked overt pronoun in *every* clause:

(1)

**Table:** 

***Ellos*** me dijeron que ***yo*** tenía anemia . . . Si ***ellos*** me dicen que ***yo*** estoy en peligro cuando ***ellos*** me entren la aguja por el ombligo, ***yo*** me voy a ver en una situación de estrés.
‘They told me that I had anemia . . . If they tell me that I am in danger when they put the needle in my belly-button, I am going to find myself in a stressful situation.’
(Toribio 2000: 319, ex. 3e).

Brazilian Portuguese (BP) has shown a similar development but going even further, to the extent that it has been categorized as a partial null subject or semi-pro-drop language (Duarte 1993; Erker and Guy 2012). This evolution seems to represent movement along van Gelderen’s (2011) subject cycle where a null subject language (NSL) can become a non-null subject language (NNSL). This is what happened in the history of French which used to allow null subjects but today requires overt pronouns (e.g. Foulet 1928). Partial null subject languages (PNSLs) would represent an intermediary stage in this cycle.1Cf. Cacoullos and Travis (2018) for evidence challenging a straightforward increase of overt subjects. The question then is what prompted this change in so many varieties of Latin American Spanish as well as BP?2A popular account has focused on the fact that phonological reduction in Caribbean Spanish has led to ambiguity in the verbal paradigm. The resulting under-specification, then, could be the catalyst for an increase in overt SPE (e.g. Lipski 1990). However, see Rosemeyer (2018) for a counter argument.


As part of a larger project to explore the hypothesis that the answer lies in their shared context of contact with the languages of enslaved Africans during the colonial period (cf. Granda 1978; Sessarego and Gutiérrez-Rexach 2017; Section 3.1), I created the historical corpus *Corpus Diacrónico del Español Latinoamericano: Edición de Sujetos* (CorDELES). Despite much discussion of the historical path of SPE in Latin American Spanish (e.g. Camacho 2013; Sessarego and Gutiérrez-Rexach 2017), a historical corpus study has yet to be carried out. Similarly, although significant contributions have been made to the study of the effects of linguistic variables such as switch-reference (Abreu 2012; Alfaraz 2015; Otheguy and Zentella 2007; Silva-Corvalán 1994; Travis 2005), priming (Cameron and Flores-Ferrán 2004; Travis 2007), person and number (Carvalho et al. 2015), TAM features (Cameron 1993; De Prada Pérez 2015), and verb class (Orozco and Hurtado 2021b; Travis 2005) on SPE, there has not been as much attention paid to inter-textual differences such as genre, particularly in the written modality.

For this reason, I apply Rosemeyer’s (2019) orality measurement to CorDELES in order to evaluate the effect of orality on SPE against the backdrop of the contact hypothesis. Concretely, I seek to first determine the relationship between orality and SPE and then assess the effects this relationship may have on the written historical corpus. The second step is accomplished by comparing the results of a mixed model analyzing the contact hypothesis with and without the orality measurement included. This study is the first (to my knowledge) that investigates the relationship between orality and SPE. Section 2 provides background on the measurement and application of orality in corpora. Section 3 first briefly summarizes the contact hypothesis that CorDELES was created to investigate – and that the orality measurement is tested in conjunction with. I then outline the building, annotation, and testing of the corpus, as well as how to quantify orality. Section 4 presents the main finding of this study: a correlation between orality and overt pronominal expression that has an intriguing diachronic component, providing far-reaching consequences for corpus methodology and SPE research. Section 5 discusses the implications of the findings and Section 6 concludes.

## 2 An introduction to orality

The issue of the difference in linguistic behavior between written and oral registers is not new. The degree to which written texts can be trusted to reflect spoken language is one of the biggest problems historical linguists face. Formality and genre differences ensure that people write differently than they speak (Biber and Conrad 2019) while prescriptivism, illiteracy, and access to publication prevent all varieties of a language from being equally represented in the written record. While the latter set of obstacles are more difficult to surmount, formality and genre are easier to account for. If we pay special attention to the types of written texts that behave most similarly to speech, then we can get a closer approximation to spoken data from historical corpora. However, genre is not stagnant (Biber and Finegan 1989). Norms can and do change over the years, especially for more artistic media like novels, poetry, and plays. ‘Apparent change’ has already been shown to affect frequencies of linguistic features in corpus studies (cf. Szmrecsanyi [2016] for the case of genitive alternation in English). In other words, superficial trends that appear to be the result of one (linguistic) factor are actually the result of a secondary (non-linguistic) factor. This concept can be applied to the inconstancy of genre style. Thus, it is not a given that a 16th century play and a 20th century play will equally reflect the spoken language of their day. This is where the ability to measure orality comes into play.

Rosemeyer (2019) investigates the development and distribution of *wh*-interrogatives in BP plays. Changes in theatre style are as relevant to SPE as to *wh*-interrogatives because both can be influenced by discourse functions that are more or less frequent depending on whether a given play relies on soliloquy, punchy dialogue, or has a narrative component. In order to make the BP corpora more comparable over time, Rosemeyer used Biber and Finegan’s (1989) “dimension of involvement” to create a functional manner of measuring orality in an easily searchable and countable way. This measurement consists of five linguistic components that either express intellectual states that tend to be conveyed in orality or depend on spatial, temporal, or discourse deixis: private verbs (*achar* ‘mean’, *pensar* ‘think’, *acreditar* ‘believe’, *crer* ‘believe’) in singular present tense, present progressive, demonstrative neuter pronouns (*isso* and *isto* ‘this’), time and place adverbs (*aqui* ‘here’ and *agora* ‘now’), and discourse markers (*né* ‘isn’t it?’, *bom* ‘well’, *pois* ‘so’, *então* ‘so’, *olha* ‘listen’). Section 3.3 reviews how these oral elements can be adapted to Spanish and applied to CorDELES. Once orality was accounted for, Rosemeyer (2019) found that many of the previously attested frequency changes for certain interrogative constructions in the 20th century were actually reflective of the changing degree of orality expressed in plays, i.e. ‘apparent’ change. Some of these increases in frequency, however, were the result of real change from below, i.e. changes in speech spreading to written plays through the most oral ones first. Before applying this orality measurement to CorDELES, an introduction to the corpus is necessary.

## 3 CorDELES

Now that the concept of orality has been elucidated, there are two main research questions that this paper seeks to address. The first is whether orality has an effect on SPE. If so, does this relationship inflate ‘apparent’ trends and/or obscure potential ones in a corpus? To answer this second research question, I test the hypotheses that overt SPE has increased (1) over time, (2) at a higher rate in Latin America than in Spain, and (3) at a higher rate in the Caribbean than South America. If a mixed-effects model finds different effects for year and region when orality is included, then orality will once again be confirmed to be an integral factor in corpus work. This section is thus dedicated to describing CorDELES, the corpus under analysis. Section 3.1 reviews the context for the creation of CorDELES which also doubles as the study orality is being applied to. Section 3.2 outlines how the corpus was built. While Section 3.3 reports how null subjects were annotated, Section 3.4 summarizes how orality was measured and quantified. Section 3.5 explains the statistical models used to analyze the results.

### 3.1 Context for the corpus

CorDELES was created to investigate the historical path that led to the higher rate of overt SPE in Caribbean Spanish as seen in (1). Considering BP has also undergone this change (Duarte 1993), their shared language contact history is likely responsible. It is crucial to remember that language contact, as well as language change, must always be viewed through the lens of language acquisition and use (Weerman 1993). It is not that two or more concrete monoliths of language come into contact, but that individual speakers learning and using new languages do. With that in mind, acquisition literature has shown that null subject systems are more difficult to acquire than non-null subject systems, particularly for L2 learners (e.g. Bini 1993; Margaza and Bel 2006; Pérez-Leroux and Glass 1999). For example, (2) is a sample from an intermediate L2 speaker who uses the overt pronoun *nosotros* ‘we’ without emphasis or focus.3The example was pulled from a written composition task where context was available.

(2)

**Table:** 

***Nosotros*** tenemos una casa en la montaña y voy cada fin de semana con amigos y amigas.
‘We have a house at the mountains and go each weekend with friends and girlfriends.’
(Margaza and Bel 2006: 95, ex. 7)

Different theories have arisen to explain why this might be. For instance, the Interface Hypothesis suggests the null subject system is difficult to acquire because it lies on the interface between multiple domains, specifically pragmatics and syntax (Sorace 2011). Alternatively, the Interpretability Hypothesis works with the concept of uninterpretability from the Minimalist framework, suggesting second language learners past the critical threshold, i.e. adults, are only able to access interpretable (that is, semantically active as well as syntactically present) features (Hawkins and Hattori 2006; Tsimpli and Dimitrakopoulou 2007; Tsimpli and Lavidas 2019). This means that when they come across uninterpretable features such as those on null subjects, they misanalyse when to use them and overcompensate with features that are interpretable: overt subjects. If it is indeed the case that null subjects are harder for adult learners to acquire, then, following Dahl’s (2004) definition of complexity as L2-difficulty, the resultant increased use of overt pronouns is an act of simplification. Thus, language contact is simplifying when the feature being learned is difficult for adults to acquire (cf. Walkden and Breitbarth 2019). Trudgill (2011) further qualifies the type of contact that would lead to strain in acquiring new systems: short-term, loose-knit, adult language learning. Those conditions perfectly describe the context for African learners of Spanish in colonial Latin America.4The other major contact scenario at the time was contact with indigenous languages, such as those within the Quechua language family. However, the circumstances were different in that contact seemed to largely take the form of individual, rather than collective, bilingualism up until the 20th century (Escobar 2011, 2012). Trudgill’s typology does not predict that this type of contact would result in simplification.


During this period, enslaved (West-)Africans were taken to the Caribbean and coastal South America (e.g. Bowser 1974). As adult second-language learners who would have had to quickly learn Spanish without consistent access to a dense network of native speakers, they would have struggled to acquire the L2-difficult null subject system, preferring overt subjects. Their children would then nativize this system. This origin is exactly what Sessarego (2013) proposes for the genesis of Afro-Hispanic Languages of the Americas (AHLAs). These varieties have been spoken by the subsequent generations of the original enslaved Africans and they demonstrate the kind of change predicted, namely an increased rate of overt pronouns. To verify this account, we need to look into the diachronic trajectory of SPE. Ideally, we would do this using historical AHLA data, but not enough exists in the written record to compare systematically over centuries. What we can do is track if this potential change might have bled into other varieties of Latin American Spanish, comparing it with AHLA data when we have it. To this end, I constructed CorDELES to attempt to find out whether overtness rates increase over the last five centuries, and if they do, if they are higher in countries with larger Afro-Hispanic populations.

### 3.2 Corpus composition

Although several western European languages are represented in parsed corpora that allow the simple extraction of null subjects, such a Spanish corpus (especially a Latin American one) does not exist. With this in mind, and due to the difficulty of counting null subjects, it was clear from the start that they would need to be annotated by hand. The simplest method would have been to use an extant corpus to export a list of every finite verb that I would have then annotated by hand. However, despite there being many excellent historical and/or dialectal Spanish corpora (e.g. CDE [Davies 2002], CORDIAM [CORDIAM n.d.], CORDE [Real Academia Española n.d.], and CDH [Real Academia Española 2013]), none provides the necessary metadata or is sufficiently POS-tagged or lemmatized so as to produce such a list. Instead, I created a corpus that I could balance and annotate directly.

CorDELES was designed to consist of 64 texts pulled from eight countries over the period 1500–1899 that are split into two genres. Table 1 shows a grid of the dimensions of the corpus as well as where each individual text (marked by an abbreviation of its title) fits into it. In the event that a prose text could not be found, one written in verse was included. Unfortunately, an extant text for every cell combination still could not be found, resulting in a shortage of seven texts. For a full listing of texts, please see Appendix A.

**Table 1:** CorDELES composition. DR is short for the Dominican Republic while LIT and DOC stand for ‘literature’ and ‘documents’, respectively. Dashes indicate a gap in the corpus, i.e. no text could be found that filled the conditions of that cell. Bolded texts are those written by a known Afro-Hispanic author. Italicized texts’ authors were born in Spain. Lastly, starred cells are written in verse.

	CARIBBEAN/CENTRAL			SOUTH AMERICAN				SPAIN
	DR	PANAMÁ	CUBA	PERÚ	COLOMBIA	BOLIVIA	VENEZUELA	
16^TH^								
LIT	ENT	*HGNI*	*HDLI*	*HNMI*	EVII*	–	*GDUI*	LAH
DOC	SDJ	*CAR*	*DRF*	*NDP*	OYC	*RVP*	*NDA*	CAN
17^TH^								
LIT	*DPHJ*	LLDP*	EDP*	CEVP*	*VDM*	–	*NHLC*	DQ
DOC	–	DLYD	LCDH	CPVV	*GNRG*	–	PR	ACRA
18^TH^								
LIT	**LIVIE**	–	PJFC*	PAD	PPYM	HVIP	*EOID*	ARJD
DOC	ASD	–	SPPH	MC	GSFB	–	ALTU	EAU
19^TH^								
LIT	GAL*	HS*	**ADUE**	**MYT**	**IHDC**	JDLR	VH	CPC
DOC	**ALD**	MPE	GDLH	**CRP**	**SYL**	ADLA	GDC	QDEV

The eight countries can be grouped into three sections on a geographical basis: Caribbean/Central America (Dominican Republic, Cuba, and Panama), South America (Peru, Colombia, Bolivia, and Venezuela), and Peninsular Spanish as a baseline. Each Latin American country was selected based on whether a known AHLA community lives there5Of course, not every country hosting a known AHLA community could be included due to the cap of eight countries I gave to keep the corpus size manageable. (Klee and Lynch 2009). AHLA populations are densest in coastal areas, reflecting where their enslaved ancestors were originally taken to. The Caribbean islands therefore hold the densest populations and should be the most likely to use overt subjects (Orozco and Guy 2008). To check the relationship between expected population level6It is very difficult to verify these population estimates as many censuses have not offered an Afro-descendant option until very recently. Even for those that do, many people do not self-identify as black or Afro-descendant. To illustrate just how challenging this can be, Colombia’s 2002 census reported a 22 % Afro-Colombian population whereas the 2005 census, just three years later, reported a drastic dip to 10.6 %. This drop likely indicates the inconsistency of the questions/options available between the censuses (DANE 2010). and pronoun usage, countries with low (Panama, Bolivia, Peru), mid (Venezuela, Colombia), and high (Cuba, Dominican Republic) Afro-Hispanic populations from the Caribbean, Central America, and South America were all represented. Admittedly, it is less than ideal to categorize Latin American varieties by country, as many of these countries already contain their own well-differentiated dialectal regions, not to mention that many of these dialects cross borders (cf. Orozco and Díaz-Campos 2016; Orozco 2018). However, the limited availability of texts made it difficult enough to fill each cell in Table 1 based only on country. Narrowing the pool to certain sub-regions would have resulted in even more gaps in the corpus. Further, information on provenance beyond country was not even available for many texts. For these reasons, the corpus was regionally delineated by country. Genre was accounted for in a rough literary versus non-literary break to balance the corpus in case it influenced SPE. Because the corpus is so small (3,773 tokens of pronominal subjects) in relation to the number of parameters and there is already a paucity of texts for certain country/century combinations, the corpus would not have been able to be adequately balanced for genre on a more fine-grained level. However, as Section 4 demonstrates, orality is a much better metric for analyzing textual effects on SPE in the corpus. Diachronically, the 16th century is included to create a baseline of the Spanish spoken in Latin America when the first Africans arrived.

The main sources for the texts were Cervantes Virtual (Biblioteca Virtual Miguel de Cervantes 1999), Biblioteca Digital Hispánica (La Biblioteca Nacional de España 2008), and the Digital Library of the Caribbean (Digital Library of the Caribbean 2004). A continuous sample of 2,000–3,000 words was randomly selected from each text. CorDELES totals 151,295 words. Some supplemental texts that were of interest but did not fit into the main corpus were also set aside, most of which will be included with data from the main corpus in the following section. The first of these texts is a transcript from an Afro-Bolivian field interview conducted in 2010 (pulled from several such transcripts provided in Sessarego [2011]). The rest are from *Cantos populares de mi tierra* and *Secundino el zapatero*, a series of poems and a play written by Candelario Obeso, an Afro-Colombian poet and playwright from the 19th century. Obeso wrote in Afro-Colombian, providing rare insight into actual historical AHLA language. These four texts also make for a fascinating comparison as they are written by the same author but split across three genres: play, poetry, and prose (pulled from the dedication). Obeso also provided translations of his poetry into standard Spanish, so any marked difference in SPE would be very telling. Each sample was then transcribed where necessary, POS-tagged by an automated tagger, and annotated by hand in XML.

### 3.3 Annotating null subjects

Part of the necessity of creating a new annotated resource rather than comparing figures from previous studies is that null subjects are very difficult to count consistently. Many judgements need to be made as to what counts as a null subject, and no two studies have taken the exact same approach, making their data tough to compare (as pointed out in de Prada 2009; Martinez-Sanz 2011; Travis 2007). In order to reliably compare frequencies from different countries, they need to have been annotated under the same guidelines. So, before looking at the data for this corpus, we must walk through some methodological decisions that needed to be made. The motivation behind these decisions was to only operate within the envelope of variation (cf. Orozco and Hurtado 2021a; Otheguy and Zentella 2007); that is, to only extract tokens for which both the presence and absence of a subject pronoun were possible. In other words, the goal was to annotate every environment where alternation was possible and only those environments.

Firstly, the focus of CorDELES is the relationship between the subject and finite verb of each clause, meaning subject pronouns of nonfinite clauses were not considered. Imperatives were also excluded due to their unique relationship with subject pronouns that allows their omission even in NNSLs. Similarly, cases of coordination were counted as only one realization of the pronoun since NNSLs are able to omit the second pronoun. E.g. *come y bebe mucho* ‘he/she eats and drinks a lot’ would count as only one null subject (that was elided for the second VP) rather than two.7Because coordinated overt pronouns are so rare in the corpus, this decision really only has an effect on the frequency of null subjects. Fixed expressions such as *hace tiempo* ‘time ago’ and *es decir* ‘that is to say’ were excluded.

Most importantly, null pronouns were tagged for animacy and referentiality because it is not possible (or at least exceptionally rare in the case of inanimacy) in standard Spanish for inanimate, non-referential/impersonal subjects to be overt. If a variety were to realize such an expletive subject overtly, it would be immediate proof that it could no longer be classified as a consistent NSL (cf. Roberts and Holmberg 2010). If this were to occur, it would be one of the final stages in the transition from an NSL to a NNSL. However, such a change is not expected to appear in the corpus (and indeed does not), so I am currently only interested in the environment where overt pronouns might *first* start overtaking null pronouns: animate, referential subjects. In addition to SPE, each clause was tagged for clause type, inversion, verbal and pronominal morphology, co-referentiality with the previous subject, priming, and interrogative status. The purpose of the present study is to analyze the potential regional and diachronic trends, so I will only consider (referential, animate) pronouns at the moment.

The biggest challenge for replicability came with some of the oldest texts in the corpus whose syntax was often difficult to parse. They contained many run-ons accompanied by lots of relative clauses that left whether a subject was coordinated or not, as well as whether it shared reference with the previous subject, unclear. In those instances, I defaulted toward creating a new null token with a different referent. I was as consistent as possible in my annotation so the texts are as comparable as can be reasonably expected, especially as I was the sole annotator. Additionally, sometimes handwritten texts could not be fully transcribed as some words were lost to bindings or smudged. If these words seemed to be the subject or finite verb of a clause, they were marked ‘unintelligible’ and excluded.

### 3.4 Measuring orality

**Figure 1:**
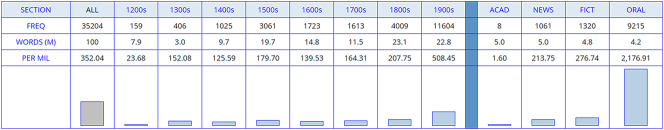
Creo ‘I think/believe’ (*n* = 35,204).

**Figure 2:**
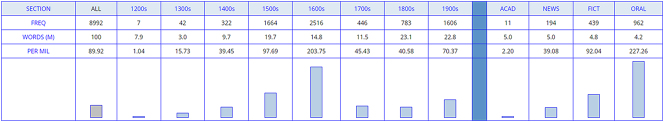
Pienso ‘I think’ (*n* = 8,992).

**Figure 3:**
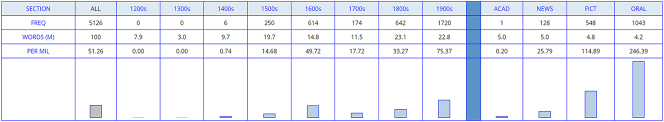
Estoy + gerund ‘I am X-ing’ (present progressive) (*n* = 5,126).

**Figure 4:**
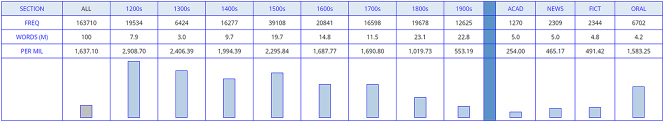
Esto ‘this’ (*n* = 163,710).

**Figure 5:**
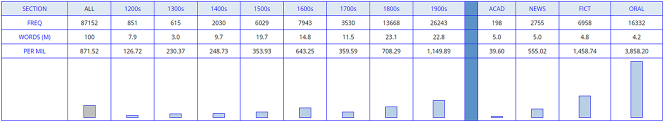
Eso ‘that’ (*n* = 87,152).

**Figure 6:**
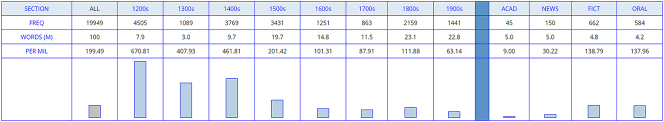
Aquello ‘that’ (*n* = 19,949).

**Figure 7:**
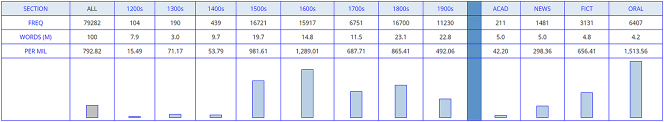
Aquí ‘here’ (*n* = 79,282).

**Figure 8:**
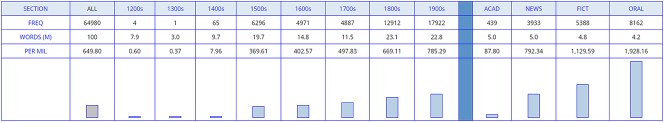
Ahora ‘here’ (*n* = 64,980).

Recall that Rosemeyer (2019) measured orality based on the presence of the private verbs *achar* ‘mean’, *pensar* ‘think’, *acreditar* ‘believe’, and *crer* ‘believe’; the present progressive, the demonstrative neuter pronouns *isso* and *isto* ‘this’; the time and place adverbs *aqui* ‘here’ and *agora* ‘now’; and the discourse markers *né* ‘isn’t it?’, *bom* ‘well’, *pois* ‘so’, *então* ‘so’, and *olha* ‘listen’. These factors were chosen to measure BP orality, but, because of its genetic and typological closeness to Spanish, they can easily be adapted for use in my corpus. To create a slightly easier number of variables to find and count, I narrowed the category of private verbs down to *creer* ‘to believe/think’ and *pensar* ‘to think’ and excluded discourse markers. The rest of the variables translate easily, leaving us with the progressive; the demonstrative pronouns *esto* ‘this’, *eso* ‘that’, and *aquello* ‘that’; and the time and place adverbs *aquí* ‘here’ and *ahora* ‘now’. The oral nature of these factors is confirmed when they are searched for by genre in the Corpus del Español (Davies 2002). Figures 1–8 each demonstrate that although the frequency of every oral marker fluctuates over time, they all consistently increase in tokens from the genre with the lowest orality to the highest: academic (ACAD) → news (NEWS) → fiction (FICT), → oral (ORAL).8These ‘oral’ texts consist of a combination of interviews and transcripts.


Once the validity of the orality measurement had been confirmed, I applied it to CorDELES. In order to quantify the gross counts in a comparable manner, I created the ORSCORE, which is derived by firstly aggregating all of the frequencies for each text, dividing them by the word count of that text, and then multiplying this figure by 100. For instance, the 19th century Spanish play CPC (the most oral text in the corpus) had 11 instances of private verbs, 6 of the progressive, 22 of demonstrative neuter pronouns, and 14 of the time and place adverbs, totaling 53 tokens. This gross frequency was then divided by CPC’s word count of 2,260, resulting in the quotient 0.02345. To make this figure more accessible, it was multiplied by 100 to reach the final ORSCORE of 2.35. On the other end of the spectrum, the 16th century Dominican court document SDJ had only 1 instance of a private verb and 0 instances of any of the other factors. Its word count was 2,008, creating the quotient 0.0005 and the final ORSCORE of 0.05.

### 3.5 Testing with mixed-effects models

A series of statistical models were run to test the effects of orality, time, and region on SPE. Each model was generated using the *lme4* package (Bates et al. 2015) in R (R Core Team 2021). A simple linear regression model (*lm*) is used to first establish the relationship between orality and SPE. In other words, a single continuous response variable (the proportion of overt subject pronouns per text) is modeled as a function of the predictor (ORSCORE). A regression line can then be plotted to represent the conditional mean formed by the model. Coefficients are the numerical values that describe this relationship and are the basis for predicted values. The differences between the model’s predicted values and the actual data points are called residuals. These residuals can be used to determine how well the model explains the data by being compared with the residuals generated by a null model. This measurement of model fit is called *R* ^2^.

The contact hypothesis, however, cannot be evaluated by a simple linear model for several reasons. First, it deals with more than one predictor (time, region, and eventually orality). One of the benefits of looking at more than one predictor in a model is that one can test for interactions. If there is an interaction between two predictors, it means that one predictor influences the response variable differently depending on the second predictor. For instance, an interaction between gender and age might mean that younger men behave differently than older men in a way that cannot be accounted for by just age or just gender. Second, at least one of these predictors is a categorical variable (region). Third, the response variable is also a categorical variable. For the linear model, SPE is turned into a continuous variable (the proportion of over subject pronouns) in order to simply visualize and analyze the relationship between orality and SPE with each text as a single data point. However, when looking at multiple predictors with numerous levels, it is better for the model to be fed each token as a single data point. Because there are only two options for the response variable (null or overt), it can be called binary. The categorical nature of the response variable means a logistic (not linear) regression needs to be run. In other words, the probability of the subject pronoun being overt instead of null is predicted. Lastly, random effects also need to be accounted for. All of the predictors previously listed are fixed effects which means that they are uniformly predictable, constant, and replicable. Random effects, on the other hand, are idiosyncratic and thus unique to each study. For instance, in an experiment, ‘participant’ would be treated as a random effect to control for differences because tokens from Participant A are more likely to pattern closely together than they are with Participant B’s tokens. For a corpus this could be anything like author, text, or newspaper. For CorDELES, the unique code for each text (docID) is specified as a random effect. Mixed-effects models are used to analyze both fixed and random effects. To fit a logistic regression to a mixed-effects model, *glmer* is used. This time, model fit is assessed by the Akaike information criterion (AIC) whereby the smaller the number is, the better the fit. The regression results for each model, including coefficient size, *p*-value, and model fit, are reported in Tables 2–4 9These tables were generated using the *stargazer* package (Hlavac 2022) and then further tweaked. in the following section.

**Table 2:** Orality-overtness linear models.

	Dependent variable:	
	Proportion of overt pronouns	
	(Total)	(No “Outliers”)
ORSCORE	0.100	0.144
	(0.023)	(0.042)
	*p* = 0.0001***	*p* = 0.002**
Constant	0.050	0.034
	(0.015)	(0.020)
	*p* = 0.003**	*p* = 0.097
Observations	37	35
*R* ^2^	0.351	0.262
Adjusted *R* ^2^	0.332	0.239
Residual Std. error	0.064 (df = 35)	0.064 (df = 33)
*F* statistic	18.906*** (df = 1; 35)	11.693*** (df = 1; 33)

*p < 0.05; **p < 0.01; ***p < 0.001.

**Table 3:** Initial mixed-effects model.

	Dependent variable:
	SPE
Scale (year)	0.458
	(0.152)
	*p* = 0.003**
Constant	−2.428
	(0.149)
	*p* = 0.000***
Observations	3,773
Log likelihood	−1,275.090
Akaike Inf. Crit.	2,556.179
Bayesian Inf. Crit.	2,574.886

*p < 0.05; **p < 0.01; ***p < 0.001.

**Table 4:** Final mixed-effects model.

	Dependent variable:
	SPE
Scale (year)	0.050
	(0.154)
	*p* = 0.744
Scale (ORSCORE)	0.030
	(0.269)
	*p* = 0.0002***
Macro_RegionAmericas	0.629
	(0.320)
	*p* = 0.049*
Scale (year):scale (ORSCORE)	−0.437
	(0.206)
	*p* = 0.034*
Constant	−2.548
	(0.276)
	*p* = 0.000***
Observations	3,773
Log likelihood	−1,268.101
Akaike Inf. Crit.	2,548.202
Bayesian Inf. Crit.	2,585.615

*p < 0.05; **p < 0.01; ***p < 0.001.

## 4 Results by text

The data in this section comes from the core of CorDELES (totaling 99,282 words): Dominican Republic, Panama, Bolivia, Colombia, and Spain. Section 4.1 establishes the correlation between orality and overt SPE. The contact hypothesis is then analyzed with orality excluded from the model in 4.2 and included in 4.3.

### 4.1 The orality correlation

**Figure 9:**
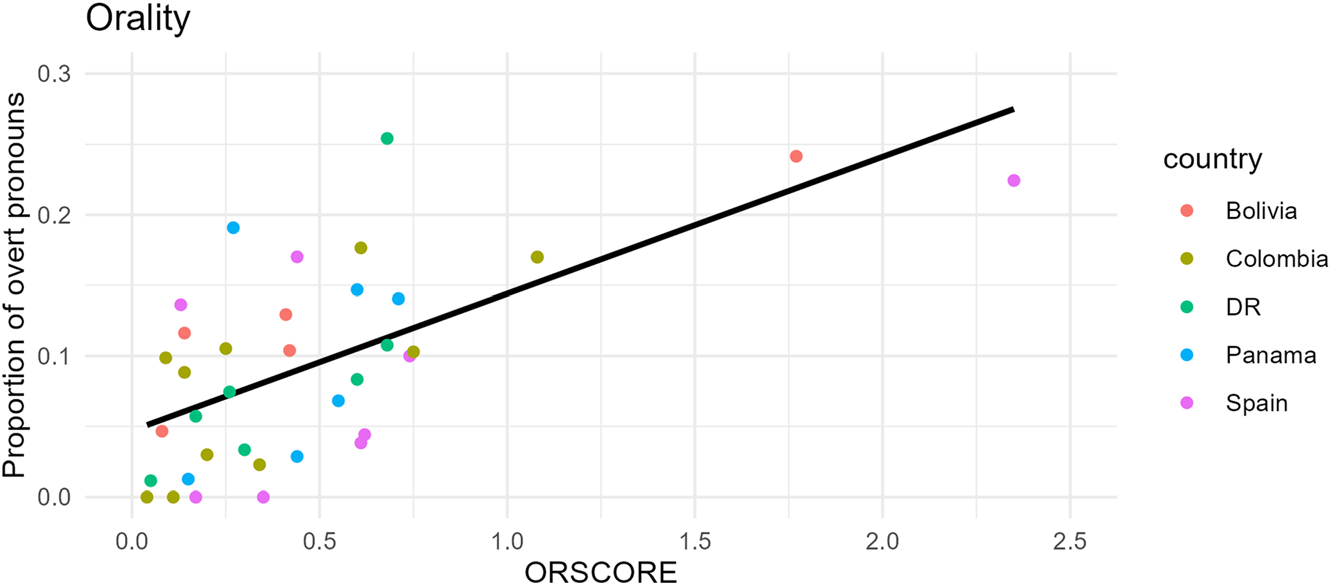
Orality and overtness regression line (*n* = 37). Each plot in this paper was created using *ggplot2* (Wickham 2016) in R (R Core Team 2021).

The ORSCORE of each text in the corpus is plotted against the proportion of overt subject pronouns in each text in Figure 9. We can see that, indeed, an orality effect is at play in the corpus data as there is a clear positive correlation between ORSCORE and the rate of overt SPE in a text. This effect is corroborated by the results of a linear model. I ran *lm* in R (R Core Team 2021), with ORSCORE as a predictor of the proportion of overt pronouns, finding a clearly significant relationship (*β* = 0.100, *p* > 0.0001, *R* ^2^ = 0.351 ± 0.02). These results are summarized in the middle column of Table 2. The *R* ^2^ score of 0.351 means that 35 % of the variance in the data can be explained by this model which is a relatively high level of explanation given all the additional factors that have been known to affect SPE (e.g. focus, switch-reference, person, etc. cf. Section 1).

**Figure 10:**
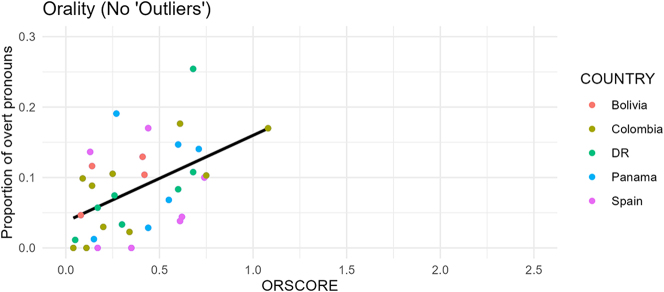
Orality and overtness regression line (‘outliers’ excluded) (*n* = 35).

There may appear to be two outliers with respective ORSCOREs of 1.77 and 2.35 in the upper right of the graph that are skewing the data. However, as the rightmost column in Table 2 shows, the *p*-value is still clearly significant (*p* < 0.002) when these two texts are excluded. In fact, the coefficient is actually larger, as illustrated by the steeper slope of the regression line in Figure 10, showing the strength of the relationship. Further, the two apparent outliers are the Bolivian oral interview (IIA) and a rapid-paced Spanish play (CPC), the two most impressionistically oral texts of the corpus, as demonstrated by examples (3) and (4). This again confirms the power of the measurement in quantifying orality.

(3)

**Table:** 

An excerpt from IIA:
Mt:^10^ ¿Y usté sabe mi nombre?
‘And you know my name?’
ss: Eeeh, sí, Torres el apellido…
‘Uh, yes, Torres the surname…’
mt: ¿Usté sabe mi nombre?
‘You know my name?’
ss: Sí, Maclobia Torres.
‘Yes, Maclobia Torres.’
mb: ¡Eso!
‘That’s it!’
ss: ¿Y el segundo apellido?
‘And the second surname?’
mt: Bediriqui, Torres Bediriqui.
‘Bediriqui, Torres Bediriqui.’

10 The speaker abbreviations stand for the interviewees Manuel Barra (MB) and Maclobia Torres (MT) and the interviewer Sandro Sessarego (SS).

(4)

**Table:** 

An excerpt from CPC:
D.’ CAR.^11^ (Con indignacion.) Está usted asesinando á mi hija.
‘(With indignation.) You are murdering my daughter’
VIDAL. Señora, usted no entiende de negocios.
‘Ma’am, you don’t understand business.’
CAROL. ¡Mamá, por Dios!
‘Mom, for God’s sake!’
D.’ CAR. Yo entiendo de todo, y cuando pasan rábanos los compro.
‘I understand everything, and when radishes come, I buy them.’
VIDAL. Yo no compro… hay tendencias á la baja.
‘I don’t buy…there are downward trends.’

11 D.’ CAR., CAROL., and VIDAL., are abbreviations for the names of the characters Doña Carolina, her daughter Carolina, and her son-in-law Señor Vidal.

Since orality has been demonstrated to have a significant effect on SPE, its influence on the rest of the corpus can now be investigated.

### 4.2 SPE over time

**Figure 11:**
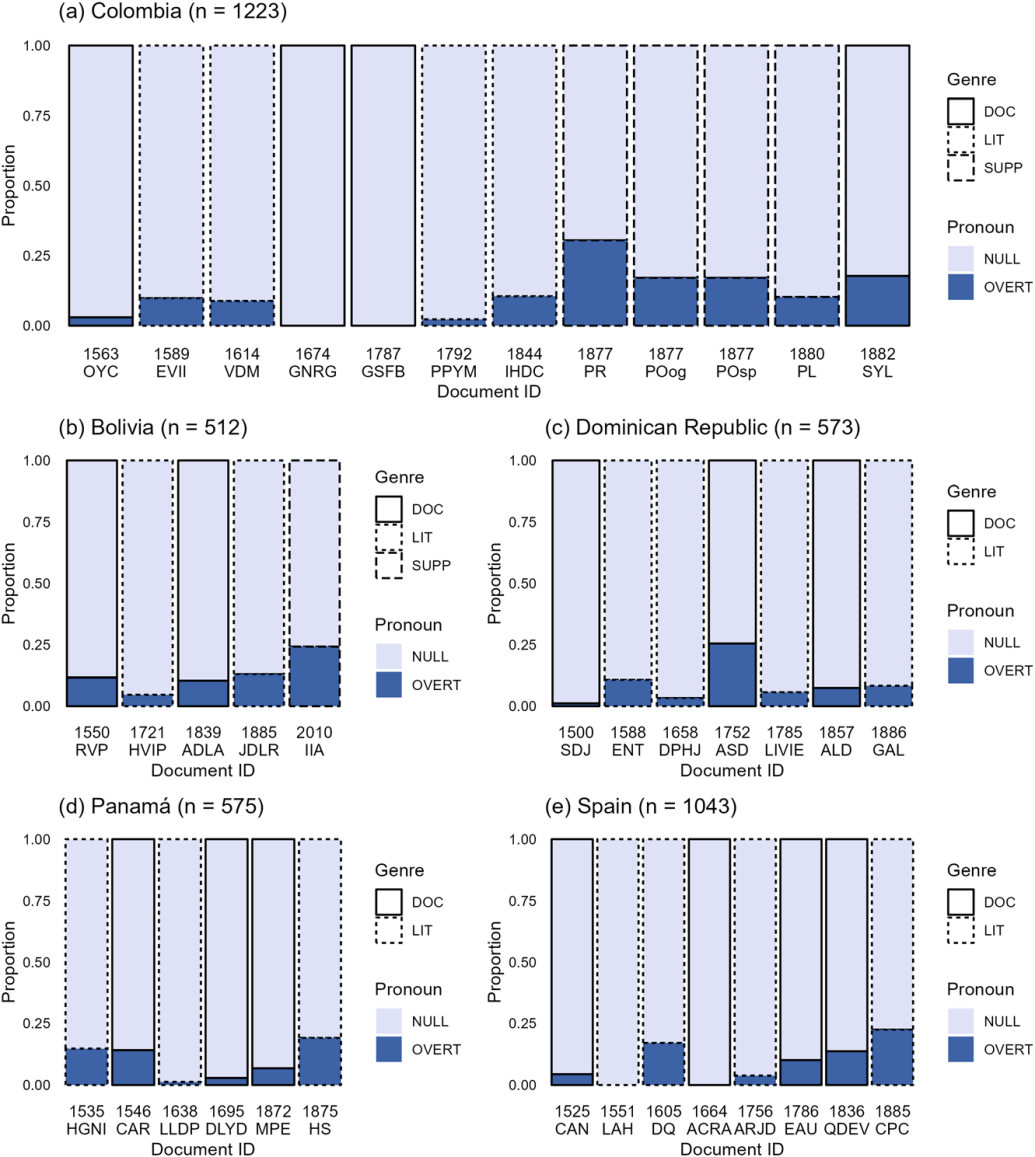
The bar charts in (a–e) give the proportion of SPE in Bolivia (*n* = 512), Colombia (*n* = 1,223), Dominican Republic (*n* = 573), Panama (n = 575), and Spain (*n* = 1,043).

First, let us look at the effects of time and region on SPE without orality being accounted for. The following bar charts (Figure 11a–e) show the proportion of null and overt subject pronouns per text. Each bar is a text identified by an abbreviation of its title, e.g. ‘ENT’ refers to the 16th century Dominican text titled *Entrémes* (cf. Table 1 and Appendix A for a full listing). These texts are ordered chronologically. The non-literary genre is illustrated with a solid border, the literary genre with a dotted border. The bars with a dashed border are the supplemental texts from Afro-Hispanic speakers.

The proportion of overt subjects appears to slightly increase in the last two centuries for most of the countries (setting aside the supplemental texts from Colombia), the only exception being the Dominican Republic which remains pretty consistent apart from ASD. That being said, these trends need to be taken with a grain of salt. Due to text scarcity, Bolivia and Panama are both missing multiple cells from this period, including the entire 17th century for Bolivia and the 18th for Panama. Further, even though there is a large gap between the last two Panamanian texts (MPE and HS), they are separated by only three years, not nearly long enough for such a rise to be the result of diachronic change alone. With that taken into consideration, there is no longer any obvious sizeable increase for Panama. This leaves Colombia as the only clear Latin American country with a noticeable increase in overt subjects. However, Spain also shows a steady increase during this period, contradicting a colonial contact account. On the other hand, since there are only a maximum of two texts per century-country combination, it is necessary to first rule out any other potential effects before drawing any conclusions.

Although there does not appear to be any genre distinction in any of the countries,12This does not necessarily suggest that the macro-genre binary was too broad as it did capture a trend elsewhere in the corpus. Inversion was also investigated, finding higher rates of SV word order in non-literary texts. This pattern was most robustly attested in the Dominican data which consistently showed higher SV order in documents by about 25 %. A mixed model confirmed this relationship, finding significance for the interaction between genre and the Dominican Republic (*p* < 0.05) and for genre overall when interactions are removed (*p* < 0.001). See McCarley (forthcoming) for further information. I ran a mixed-effects logistic regression model using *glmer* from the *lme4* package (Bates et al. 2015) (cf. Section 3.5) to see if it could pick up any underlying patterns that are unclear from visual inspection of the bar charts. SPE was run as a binary dependent variable while country, year (z-scored), and genre (literary vs. non-literary) were fixed effects, and docID was a random effect.13The total percentage of overt subject pronouns is 12.9 % (432/3,773 observations).Number of occurrences per country: Bolivia, *n* = 512; Colombia, *n* = 1,070; Dominican Republic, *n* = 573; Panama, *n* = 575; Spain, *n* = 1,043.Number of occurrences per genre: literary, *n* = 2,522; non-literary, *n* = 1,251. Two other regional groupings with fewer levels were also run: region (Caribbean, South America, Spain) and macro-region (Spain vs. the Americas). Over 20 versions of the model were run, testing for every possible combination of fixed effects with and without interactions. Some iterations of the model did not have enough data to converge. These were versions with multiple interactions or interactions with country due to the numerous levels involved. Of the models that converged, year was the only predictor to ever emerge significant (*β* = 0.458, SE = 0.152, *p* < 0.003). Similarly, the model with the best fit according to AIC scores was the model that only specified year as a fixed effect. These results are reported in Table 3. In sum, a positive relationship between year and overt SPE was found, i.e. pronouns are more likely to be overt over time.

Even though genre does not have a significant effect in the model, something similar to genre seems to be influencing the data. The texts with some of the highest overt SPE rates also show high levels of dialogue: ASD is an 18th century Dominican court document that transcribes the testimony of witnesses describing events; HS is a 19th century Panamanian series of poems that frequently address a specific audience in 2nd person; the poetry from the supplemental 19th century Afro-Colombian text CPMT similarly uses frequent dialogue;14The prose text actually has the highest rate of overtness of the Obeso texts. See Section 5 for a discussion of these genre differences. IIA is the 21st century transcript of an Afro-Bolivian oral interview; DQ is a sample from the 17th century Spanish novel *Don Quixote* with a lot of dialogue; and CPC is the 19th century Spanish play with a lot of rapid back-and-forth. Given IIA and CPC also had the two highest ORSCOREs in the corpus (cf. Section 4.1), orality seems to be the key to this pattern. Before verifying orality’s role by introducing it into the model, it is important to rule out other potential sources for this discourse-heavy trend.

All of these texts seem to have rich environments for discourse-switching in which the referent for each subject frequently changes clause to clause. Given that switch-reference has a well-known effect on SPE (e.g. Abreu 2012; Alfaraz 2015; Otheguy and Zentella 2007; Silva-Corvalán 1994; Travis 2005), I had already tagged each pronoun for whether it shares reference with the previous subject token or not (cf. Section 2). However, co-reference levels did not correlate with overt SPE in any of the countries. If the high discourse levels are not a function of switch-reference, then maybe genre was on the right track and this pattern is the result of textual differences.

The next logical step following genre would be to look at subgenre/text-type, specifically plays since they are intuitively expected to have the highest levels of dialogue. There are four plays in the corpus (ENT from 16th century Dominican Republic, ARJD from 18th century Spain, CPMTpl from 19th century (Afro-)Colombia, and CPC from 19th century Spain), and they make up a wide range of overt pronominal rates: 11 %, 3.8 %, 10 %, and 22 %, respectively. This variation is particularly striking considering the lowest and highest of the plays’ frequencies are from the same country and only a century apart. Recall, however, that Rosemeyer (2019) points out that it is misleading to assume that all plays should behave uniformly because they may differ in their degree of orality. For BP, stylistic change in the level of orality represented in plays was creating ‘apparent’ change in the diachronic patterning of certain *wh*-interrogatives (cf. Section 2). In order to evaluate whether orality is doing the same thing to SPE rates in this corpus, I use the measurement of orality outlined in Section 3.4 to analyze its effects in CorDELES in the next section.

### 4.3 An interaction between orality and time

In Section 4.1 I discovered a relationship between orality and overt SPE in CorDELES. In the same corpus, I also found a significant correlation between year and overt SPE in Section 4.2. The question follows as to whether the former pattern is a real trend responsible for an artificial inflation of the latter. This would mirror Rosemeyer’s (2019) findings but be detrimental for the ability of this corpus to accurately investigate the contact hypothesis. There is another possible eventuality in which the inclusion of orality would give the mixed-effects model more clarity, revealing nuances to the relationships SPE has with year and region that could not be captured with the orality noise unaccounted for. Recall that the *R* ^2^ value for the linear model run in Section 4.1 was only 0.351, meaning only 35 % of the variance in the data can be explained by the relationship between orality and proportion of overt pronouns. Despite the strong relationship in Figure 9, there is still clearly a high degree of variation in overt SPE rates for texts of a similar ORSCORE. For instance, the three texts DLYD (from 17th century Panama), ADLA (from 19th century Bolivia), and DQ (from 17th century Spain) all had similar ORSCOREs of 0.48, 0.42, and 0.44, respectively, whereas their proportion of overt subjects varied appreciably with rates of 2.9 %, 10 %, and 17 %, respectively. Much of this variation is likely due to linguistic influences such as pragmatic conditioning (e.g. focus, emphasis, contrast), but it also leaves room for the possibility that there are underlying regional (and perhaps further diachronic) trends in the corpus still to be uncovered. The next step is to try and tease those apart from this robust orality effect. Either way,15There is, of course, a third option where the inclusion of orality adds nothing new to the results of the mixed model. Given the robust correlation between orality and overt SPE (cf. Section 4.1), this seems unlikely. the inclusion of orality in the model is paramount to get an accurate analysis of the diachronic and regional trends in CorDELES.

The best way to do this is to include orality as a predictor in the mixed model. *Glmer* from *lme4* (Bates et al. 2015) was again run in R (R Core Team 2021) with SPE as a binary dependent variable and docID as a random effect. Again, every possible combination of predictors and interactions that included orality was run. Once more, the number of tokens did not span far enough to cover iterations with multiple interactions or interactions with country. Predictably, every model that did converge found ORSCORE to be significant. What is new is that certain combinations found each regional grouping and/or its interaction with ORSCORE to be significant. Of all these models, the one with the lowest AIC (i.e. the model that best described the spread of data) had ORSCORE, year (both z-scored), and macro-region (Spain vs. the Americas) as fixed effects with an interaction between year and ORSCORE.16At first, country was included as a fixed effect again, but it was not found to be significant. Neither was region (Caribbean vs. South America vs. Spain). The number of occurrences for macro-region are 1,043 for Spain and 2,730 for the Americas. The results of this model are summarized in Table 4 which reveals that this final model returns three significant findings. First, as expected, ORSCORE is the most significant predictor with an effect size of 1.030 (this is the coefficient that determines the slope of the regression line, cf. Section 3.5) and a *p*-value less than 0.001.

**Figure 12:**
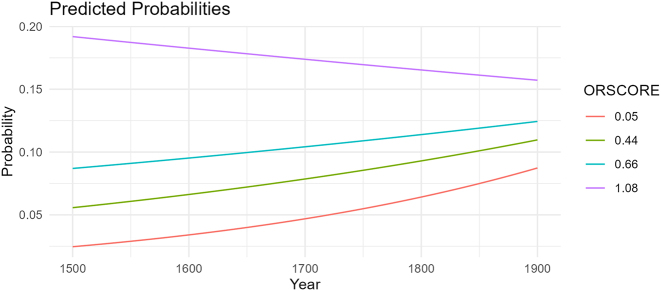
Predicted SPE across ORSCOREs over time.

Second, now that orality is included in the model, year is no longer significant on its own; however, its interaction with orality is (*β* = −0.43702, SE = 0.20634, *p* < 0.034177). The negative coefficient means that for every year that increases, the effect of orality on overtness decreases. In other words, the texts are becoming more oral over time, but their levels of overtness are also increasing beyond what orality can explain on its own. This trend is clear with the use of *plogis* in R (R Core Team 2021) to calculate the predicted probabilities for this interaction given a specific ORSCORE. In other words, one can provide a start year, end year, and precise ORSCORE and the function will return the proportion of overt SPE that the model predicts for the given orality level for each year. For instance, for the median ORSCORE of 0.45, the rate of overt SPE is predicted to increase from 5.7 % to 11 % from 1,500 to 1,900. These predictions are plotted in Figure 12. Note that the gaps between lines become smaller over time, narrowing toward each other. This is the effect of the interaction between orality and year at play: the difference in SPE rates between orality levels is not as large in 1,900 as it is in 1,500. Surprisingly, while the minimum (0.05), median (0.45), and mean (0.66) ORSCOREs all show a steady increase over time, the maximum ORSCORE (2.35)17There is a case to be made for excluding these “outliers” mentioned earlier since they are more than two standard deviations away from the mean of the dataset. However, the text with the third highest ORSCORE (CPMTpoOG, 1.08) is not, and the predicted proportions still decrease at that level. Interestingly, the model without interactions shows a predicted increase regardless of how high the ORSCOREs reach. predicts a decrease from 79 % to 30 %. This prediction does not match the actual data as none of the SPE proportions pass 30 % to begin with. This discrepancy is likely because there are only three texts with ORSCOREs above 1.00, two of which are more than double and triple the mean ORSCORE. Since the model has so little data for such high ORSCOREs (and none before the year 1877), it likely cannot make accurate diachronic predictions for ORSCOREs that large. This is not to say that the interaction is not present for high ORSCOREs, just that they are more likely to pattern like the rest of the ORSCOREs affected by the interaction. In other words, the overt SPE rates predicted for an ORSCORE of 1.08 would increase over time,18Or potentially stay stagnant if the ‘apparent’ change account is correct, see Section 5 for more details. but the gap between them and the rates for a 0.66 ORSCORE would still shrink.

In addition to the interaction between year and orality, an effect of macro-region has also come out significant in the new model (*β* = 0.62880, SE = 0.31997, *p* < 0.049389). This means that the American data favors overt SPE more than Spain. Although this is hardly surprising as the dialectal differences in SPE have been well-attested (thus motivating the present study, cf. Section 1), it was troubling that the original model did not find a difference. That being said, while the effect is consistent with the regional half of the contact hypothesis, it does not fully answer it. The contact hypothesis specifically predicts an interaction between macro-region and year or between year, orality, and macro-region. This would have shown that the two regions do not just behave differently, but came to behave differently diachronically, i.e. after the catalyst of language contact in the Americas. However, neither of those models found these interactions significant. Indeed, the three-way interaction model did not find anything significant at all, even orality. Their AIC scores were accordingly higher than the final model’s, meaning they did not explain the data’s distribution as well.

In sum, the new model proves that there is a diachronic effect in the corpus which is tied to orality. It has also demonstrated that a regional trend seems to have been obscured by orality, something not apparent in either Figure 11a–e or the initial models. The following section will flesh out the implications of these findings and the role of orality in general.

## 5 Discussion and further directions

The goal of this paper was to first establish whether orality had an effect on SPE, and if so, evaluate how this relationship affects a corpus. This corpus was CorDELES which was built to investigate a language contact origin for the overproduction of overt SPE in certain varieties of Latin American Spanish. Orality was in fact found to positively correlate with the proportion of overt SPE per text. While its inclusion in the mixed-effects model used to analyze the corpus does not fully support the contact hypothesis, it does reveal a previously obscured distinction between Spain and the rest of the data as well as a curious interaction between orality and time. Specifically, this interaction means that the effect orality has on SPE has lessened over time.

There are five major insights from these orality results in CorDELES. Firstly and most broadly, these results support Rosemeyer (2019) and Szmrecsanyi’s (2016) arguments that environmental or ‘apparent’ change in historical corpus data need to be accounted for. It is easy to mistake artificial stylistic changes for what appear to be actual diachronic changes, but the trappings of a genre are not always constant and cannot be assumed to be so. That genre is not a direct correlate for orality levels is made clear by the four Obeso texts. Impressionistically, the play should be the most oral, yet it is the poetry whose orality is so high that it surpasses the bulk of the data’s. Current and future corpus study needs to make more of an effort in accounting not only for genre but also orality in order to better balance corpora over time. Secondly, these results also build on Rosemeyer’s pinpointing of fluctuating orality as this kind of deceptive environmental change since the effect of year appears to be more significant before orality is taken into account. However, ‘unapparent’ change also appears to be covered up by orality as a macro-region distinction between Spain and the Americas only emerges significant once orality is included in the model. This finding also affects the diachronic increase. Before orality, the increase did not discriminate between Spain and the rest of the data which was a problem for the contact hypothesis. However, the effect of orality seems to have created an ‘apparent’ but not genuine diachronic rise in overtness in Spain. This is supported by the fact that the most recent text from Spain (CPC) is the text with the highest orality out of the entire corpus. In other words, in addition to finding artificial patterns caused by changes in orality, I also initially found no discernable regional patterns *because of* the correlation between orality and overt SPE. More practically, this study also supports the validity of Rosemeyer’s orality measurement and encourages its adaptation for other corpus work. Thirdly, this positive correlation speaks to the reliability of historical null subject research. It suggests that pronouns are more likely to be realized as overt in oral speech than written texts. While this is not an entirely unexpected development, it nonetheless forms a chink in the already frayed armor of using historical corpora to track spoken change. However, all hope is not lost.

An unexpected finding emerged in the form of an interaction between orality and year. It is tempting to interpret this interaction as another orality-led case of ‘apparent’ but not real change, i.e. the increase in overt SPE over time is really just an increase in orality over time. Written texts, then, would have undergone a stylistic change to more closely reflect spoken language just as was the case for some of the patterns for BP *wh*-interrogatives (Rosemeyer 2019). In other words, speech would have always used a consistent SPE rate and the style shift towards more oral texts simply represents the written register catching up. At least, this would be the case for the time span of the corpus which ends in 1900, since the frequently attested overproduction of overt SPE in the Caribbean Spanish spoken today (cf. Section 1) means change needs to have occurred at some point. Crucially, this means that this ‘apparent’ change account is not inconsistent with the contact hypothesis. The latter change may have simply taken longer to spread to the (mostly standard) Spanish used when writing such that 1900 is too early a cut-off point for the corpus. Indeed, this timeline would parallel the change in BP which only really began in the mid-19th century (Duarte 1993). Alternatively, this attested rise might be a change from below. Specifically, the overproduction of overt SPE by L2 learners and their subsequent generations would first spread to the speech of others. In written texts, this change would subsequently be captured in the most oral texts first. This type of change was also found in Rosemeyer’s (2019) study.

Should a story of colonial contact-initiated simplification over time be borne out, the slowness of the increase in overt pronouns could be due to the underlying dynamics of change in null subjects. Kauhanen (2022), following Yang (2002), created a model to predict the behavior of L1 and L2 variational learners, using the case study of null subject acquisition in Afro-Peruvian Spanish. According to his model, the proportion of L2 speakers in a population that the critical threshold would require for full simplification (100 % overt subjects) is 0.9 (on a scale of 0–1.0). Estimates of the actual historical proportion for Afro-Peruvians vary between 0.2 and 0.6, nowhere close to the necessary proportion. As this number is exceedingly high and difficult to reach, it is unlikely that any of the countries under study would have reached this threshold, explaining why the increase in overtness (or partial simplification) observed in this corpus is so slow.

Another potential explanation for the interaction is that a new factor that influences SPE rates emerged at some point over the corpus. As it gained influence over time, the other factors that determined SPE (e.g. orality) lost some of their influence in turn. For the contact hypothesis, this new factor would be the influx of L2 learners and their subsequent generations. This third account is not borne out by the corpus results at present, since a regional interaction with time could not be found. The extension of CorDELES to Peru, Venezuela, and Cuba may shed more light on the matter. Regardless, the investigation of the contact hypothesis in a corpus that is controlled for orality (i.e. a corpus where all texts are of similar orality levels) would be ideal. If overt SPE still increases in the same time period covered by CorDELES, the ‘apparent’ change account is false and the contact hypothesis is partially supported. For it to be fully supported, the American data would also have to behave distinctly from Spain in a meaningful way.

This leads to the one last loose end that needs to be tied up. The story for simplification initiated by the origin of AHLAs is broadly supported by the significance of Spain versus the Americas in the final model. However, an interaction with time could not be found, even when accounting for orality. It is possible that the model simply does not have enough data yet to capture any such distinctions. This can be solved by extending CorDELES to include data from Cuba, Peru, and Venezuela, feeding more data to the mixed models. A secondary purpose in extending the corpus now is to also confirm this orality effect in the rest of the data. The limitation of only two texts per country-century combination likely also makes it difficult to disentangle country versus individual text-level variation. The answer there would be to at least double the size of CorDELES. However, it was difficult to fill cells with just two texts so this could result in an even heavier skew in the corpus. What is more plausible is that a region-based (Caribbean, South America, Spain) effect may still be borne out.

## 6 Conclusions

This paper first sought to address whether there is a correlation between orality and SPE. Indeed, a significant positive correlation between orality and overt SPE was found. The second research question asked whether the relationship between orality and SPE affected the analysis of a corpus, and the answer is a resounding yes. This outcome was achieved by assessing the results of a mixed model evaluating the theory that nontargetlike language learning by enslaved Africans during the colonial period led to the increased use of overt subject pronouns in communities throughout Latin America (Sessarego 2013; Sessarego and Gutiérrez-Rexach 2017). Concretely, the hypothesis was that overt SPE should increase over time at a higher rate in the Caribbean than South America and higher still than in Spain. The results of the model changed significantly once orality was included, uncovering a regional distinction between Spain and the Americas and an interaction between orality and time. Several possible explanations for this interaction were put forth, all of which encourage taking orality in account in larger corpora to attempt to replicate and confirm this effect. Similarly, although the contact hypothesis could not be fully supported by the corpus as stands, the results found are not inconsistent with a history of language contact either. The results of this study have discovered a relationship between SPE and orality that has far-reaching consequences for both future corpus work and pronominal subject study.

## Appendix A

**Table:**

Text	Shortened title	Country	Year	Author	Source
dr16ent	*Entremés*	DR	1588	Cristóbal de Llerena	CervantesVirtual
dr16sdj	*Sobre lo de la justicia*	DR	1500	–	SALALM
dr17dphj	*Discurso politico histórico jurídico*	DR	1658	Montemayor y Córdoba de Cuenca, Juan Francisco	BDH
dr18livie	*La idea del valor de la Isla Española*	DR	1785	Antonio Sánchez Valverde	BDH
dr18asd	*Autos seguidos en la Audiencia de Santo Domingo*	DR	1752	–	BDH
dr19gal	*Galaripsos*	DR	1883–89	Gaston Fernando Deligne	Print
dr19ald	*A los Dominicanos*	DR	1857	Buenaventura Báez	dLOC
pa16hgni	*Historia general y natural de las Indias*	Panamá	1535	Gonzalo Fernández de Oviedo y Valdés	Cervantes Virtual
pa16car	*Carta del Adelantado Pascual Andagoya*	Panamá	1546	Pascual de Andagoya	BDH
pa17lldp	*Llanto de Panamá a la muerte de don Enrique*	Panamá	1638	Mateo Ribera et al.	Print
pa17dlyd	*Discurso legal y defensa*	Panamá	1695	Diego Ladrón de Guevara, Obispo de Quito	BDH
pa19hs	*Hojas Secas*	Panamá	c.1875	Amelia Denis de Icaza	La Revista Cultural Lotería
pa19mpe	*Mensaje del Presidente del Estado*	Panamá	1872	Buenaventura Correoso	La Revista Cultural Lotería
cu16hdli	*Historia de las Indias*	Cuba	1561	Bartolomé de las Casa	Cervantes Virtual
cu16drf	*Declaración de Rodrigo de Figueroa*	Cuba	1520	Rodrigo de Figueroa	CHARTA
cu17edp	*Espejo de paciencia*	Cuba	1608	Silvestre de Balboa	Print
cu17lcdh	*La ciudad de la Habana,*	Cuba	1626–1630	–	BDH
cu18pjfc	*El príncipe jardinero y fingido Cloridano*	Cuba	1739	Santiago de Pita	Cervantes Virtual
cu18spph	*El sesquicentenario del papel periódico de la Havana*	Cuba	1790	–	dLOC
cu19adue	*Autobiografia de un esclavo*	Cuba	1839	Juan Francisco Manzano	OMEGALFA
cu19gdlh	*Gaceta de la Habana*	Cuba	1852	–	University of Miami Digital Collections
pe16hnmi	*Historia natural y moral de las Indias*	Perú	1591	José de Acosta	Cervantes Virtual
pe16ndp	*Noticia del Perú*	Perú	1533	Miguel de Estete	Cervantes Virtual
pe17cevp	*Coloquio entre la vieja y Periquillo sobre una procesión celebrada en Lima*	Perú	1645–1698	Juan del Valle y Caviedes	Cervantes Virtual
pe17cpvv	*Carta de Pedro Vázquez de Velasco*	Perú	1665	Pedro Vázquez de Velasco	BDH
pe18pad	*Paulina o el amor desinteresado*	Perú	1725–1803	Pablo de Olavide	Cervantes Virtual
pe18mc	*Montemar Cartas*	Perú	1761–1765	Pedro Carrillo de Albornoz y Bravo de Lagunas	University of Illinois Library
pe19myt	*Mujer y tigre*	Perú	1890	Ricardo Palma	Cervantes Virtual
pe19crp	*Cartas de Ricardo Palma*	Perú	1893–1897	Ricardo Palma	Cervantes Virtual
co16evii	*Elegías de varones ilustres de Indias*	Colombia	1589	Juan de Castellanos	BDYCL
co16oyc	*Ordenanzas y comisiones para el Reino de Nueva Granada y Obispado de Quito*	Colombia	c.1563	–	BDH
co17vdm	*Viaje del mundo*	Colombia	1614	Pedro Ordóñez de Ceballos	Cervantes Virtual
co17gnrg	*Genealogias del Nuevo Reino de Granada*	Colombia	1674	Juan Flórez de Ocariz	Cervantes Virtual
co18ppym	*Pensamientos políticos y memoria sobre la población del Nuevo Reino de Granada*	Colombia	1792	Pedro Fermin de Vargas y Sarmiento	Cervantes Virtual
co18gsfb	*Gazeta de Santa Fe de Bogotá*	Colombia	1787	–	Cervantes Virtual
co19ihdc	*Ingermina o la hija de Calamar*	Colombia	1844	Juan José Nieto Gil	Universidad de Antioquia Repositorio de proyectos
co19syl	*Sombra y Luz*	Colombia	1874–1899	Luis Antonio Robles Suárez	Universidad del Rosario Repositorio institucional
bo16rvp	*Relacion al Virrey del Peru*	Bolivia	1500–1600	–	BDH
bo18hvip	*Historia de la villa imperial de Potosi*	Bolivia	1705–1736	Bartolomé Arzáns de Orsúa y Vela	Brown University Library
bo19jdlr	*Juan de la Rosa*	Bolivia	1885	Nataniel Aguirre	Cervantes Virtual
bo19adla	*El Atalaya de los Andes*	Bolivia	1839	Bernardino Palacios	Cervantes Virtual
ve16gdui	*Geografía y descripción universal de las Indias*	Venezuela	1574–1598	Juan López de Velasco	Cervantes Virtual
ve16nda	*Noticias de America*	Venezuela	1500s	Pedro de Mendoza	Cervantes Virtual
ve17nhlc	*Noticias historiales de las conquistas de Tierra Firme en las Indias Occidentales*	Venezuela	1626	Fray Pedro Simón	Lau Haizeetara
ve17pr	*Pesquisa realizada*	Venezuela	1693	Juan de los Santos	Saber UCV
ve18eoid	*El Orinoco ilustrado y defendido*	Venezuela	1745	José Gumilla	Internet Archive
ve18altu	*Arca de letras y theatro universal*	Venezuela	1783	Fr. Juan Antonio Navarrete	Cervantes Virtual
ve19vh	*Venezuela heroica*	Venezuela	1881	Eduardo Blanco	Internet Archive
ve19gdc	*Gazeta de Caracas*	Venezuela	1808	–	Cervantes Virtual
sp16lah	*Libro de la anathomía del hombre*	Spain	1551	Bernardino Montaña de Monserrate	TeXTReD
sp16can	*Cartas del abad de Nájera al emperador Carlos V*	Spain	1525	el abad de Nájera	BDH
sp17dq	*Don Quijote*	Spain	1605	Miguel de Cervantes	Project Gutenberg
sp18arjd	*Abeja racional en el jardin de los donayres*	Spain	1756	Pedro Jiménez y Fernández	BDH
sp18eau	*El apologista universal*	Spain	1786	Pedro Centeno	BDH
sp19cpc	*4 por 100 :comedia en un acto y en prosa*	Spain	1885	Emilio Sánchez Pastor	BDH
sp19qdev	*Quedan declarados en venta todos los bienes que hubiesen pertenecido a las Comunidades y Corporaciones religiosas extinguidas*	Spain	1836	–	Filosofía en español
